# Thyroid hormones induce browning of white fat

**DOI:** 10.1530/JOE-16-0425

**Published:** 2016-12-02

**Authors:** Noelia Martínez-Sánchez, José M Moreno-Navarrete, Cristina Contreras, Eva Rial-Pensado, Johan Fernø, Rubén Nogueiras, Carlos Diéguez, José-Manuel Fernández-Real, Miguel López

**Affiliations:** 1 Department of PhysiologyCIMUS, University of Santiago de Compostela-Instituto de Investigación Sanitaria, Santiago de Compostela, Spain; 2 CIBER Fisiopatología de la Obesidad y Nutrición (CIBERobn)Santiago de Compostela, Spain; 3 Department of DiabetesEndocrinology and Nutrition, Hospital de Girona ‘Dr Josep Trueta’, Institut D’investigació Biomèdica de Girona (IdIBGi) and University of Girona, Girona, Spain; 4 Department of Clinical ScienceKG Jebsen Center for Diabetes Research, University of Bergen, Bergen, Norway

**Keywords:** AMPK, browning, thyroid hormones, white adipose tissue

## Abstract

The canonical view about the effect of thyroid hormones (THs) on thermogenesis assumes that the hypothalamus acts merely as a modulator of the sympathetic outflow on brown adipose tissue (BAT). Recent data have challenged that vision by demonstrating that THs act on the ventromedial nucleus of the hypothalamus (VMH) to inhibit AMP-activated protein kinase (AMPK), which regulates the thermogenic program in BAT, leading to increased thermogenesis and weight loss. Current data have shown that in addition to activation of brown fat, the browning of white adipose tissue (WAT) might also be an important thermogenic mechanism. However, the possible central effects of THs on the browning of white fat remain unclear. Here, we show that 3,3′,5,5′ tetraiodothyroxyne (T_4_)-induced hyperthyroidism promotes a marked browning of WAT. Of note, central or VMH-specific administration of 3,3′,5-triiodothyronine (T_3_) recapitulates that effect. The specific genetic activation of hypothalamic AMPK in the VMH reversed the central effect of T_3_ on browning. Finally, we also showed that the expression of browning genes in human WAT correlates with serum T_4_. Overall, these data indicate that THs induce browning of WAT and that this mechanism is mediated via the central effects of THs on energy balance.

## Introduction

Thyroid hormones (THs; 3,3′,5,5′ tetraiodothyroxyne or T_4_ and 3,3′,5-triiodothyronine or T_3_) exert important biological actions, not only modulating the development and growth but also regulating metabolism and energy balance (Brent 2012, Warner & Mittag 2012, Lopez *et al*. 2013). Impaired function of the thyroid gland, by either hyperthyroidism or hypothyroidism, leads to alterations in metabolism and energy homeostasis. Hyperthyroidism is associated with an increase in the metabolic rate and the patients suffering from this condition undergo body weight loss, despite increased food intake; quite the opposite, hypothyroid patients show lowered metabolic rate and reduced food intake (Brenta *et al*. 2007, Kaptein *et al*. 2009, Pearce 2012).

THs are key regulators of thermogenesis, which represents a major component of the energy expenditure in homeothermic (‘warm-blooded’) animals (Cannon & Nedergaard 2004, Silva 2006). In mammals, including humans, thermogenesis occurs mainly in the brown adipose tissue (BAT) (Cannon & Nedergaard 2004, Silva 2006, von Ballmoos *et al*. 2009). THs act on brown adipocyte thermogenesis by increasing the stimulatory action of norepinephrine (NE), as well as enhancing the cAMP-mediated acute rise in *ucp1* gene expression (Bianco *et al*. 1988, Silva 2006, Ribeiro *et al*. 2010). The existence of central effects of THs in the regulation of BAT thermogenesis was proposed long time ago (Nedergaard *et al*. 1997). Recent evidence from our group has also shown a homeostatic link between the central effects of THs on hypothalamic AMP-activated protein kinase (AMPK), sympathetic tone and UCP1 expression in BAT (Lopez *et al*. 2010, Alvarez-Crespo *et al*. 2016).

Over the last years, accumulating evidence has demonstrated that activation of beige/brite (‘brown in white’) adipocytes in the white adipose tissue (WAT), a process known as browning (Fisher *et al*. 2012, Cohen *et al*. 2014, Nedergaard & Cannon 2014, Contreras *et al*. 2016*b*), is responsible for a significant increase in total energy expenditure (Shabalina *et al*. 2013). Thus, stimulation of browning has therapeutic potential to promote body fat reduction (Yoneshiro *et al*. 2013, Beiroa *et al*. 2014). Several mechanisms have been proposed for WAT browning (Villarroya & Vidal-Puig 2013, Nedergaard & Cannon 2014), including prolonged cold exposure (Loncar *et al*. 1986), adrenergic activation (Cousin *et al*. 1992, Ghorbani *et al*. 1997, Cao *et al*. 2011) and also thyroid hormone receptor (TR) agonism (Lin *et al*. 2015, Alvarez-Crespo *et al*. 2016). However, the role of central THs in the control of WAT browning remains unclear.

The aim of this study was to investigate the role of central THs on the browning of WAT and the mechanisms behind this action. Our data show that peripherally induced hyperthyroidism promoted browning of white fat and that this effect is recapitulated by central and specific administration of T_3_ in the ventromedial nucleus of hypothalamus (VMH), via a mechanism dependent of AMPK. Notably, we also demonstrate that the expression of browning markers in WAT correlates with serum T_4_ levels in humans. Thus, in addition to the well-known effects of central THs on BAT (Lopez *et al*. 2010, Alvarez-Crespo *et al*. 2016), our data indicate an additional mechanism by which central THs influence energy expenditure, namely browning of WAT.

## Material and methods

### Animals

Male Sprague–Dawley rats (200–250 g; Animalario General USC, Santiago de Compostela, Spain) were housed on a 12-h light (08:00–20:00), 12-h darkness cycle, in a temperature and humidity controlled room and maintained with chow (STD, *SAFE A04:* 3.1% fat, 59.9% carbohydrates, 16.1% proteins, 2.791 kcal/g; Scientific Animal Food & Engineering; Nantes, France) and water *ad libitum*. For all the procedures, the animals were individually caged and used for experimentation 7 days later. During all experimental approaches, animals and their respective food intake and body weight were monitored every day. The experiments were performed in agreement with the International Law on Animal Experimentation and were approved by the USC Ethical Committee (Project ID 15010/14/006).

### Patients

A group of 163 (80 visceral, vWAT and 83 subcutaneous, sWAT) white adipose tissues from participants were analyzed (Table 1). These participants were recruited at the Endocrinology Service of the Hospital of Girona ‘Dr Josep Trueta’. All subjects were of Caucasian origin and reported that their body weight had been stable for at least three months before the study. Subjects were studied in their post-absorptive state. They had no systemic disease other than obesity and all were free of any infections in the previous month before the study. Liver diseases (specifically tumoral disease and HCV infection) and thyroid dysfunction were exclusion criteria. All subjects gave written informed consent, validated and approved by the Ethics committee of the Hospital of Girona ‘Dr Josep Trueta’, after the purpose of the study was explained to them.

**Table 1:** Anthropometric and clinical parameters.

Sex (men/women)†	14/69
Age (years)	45.24 ± 10.5
BMI (kg/m2)	42.3 ± 8.4
Fasting glucose (mg/dL)	95.5 (85.5–112.2)*
Total cholesterol (mg/dL)	186.3 ± 30.3
HDL cholesterol (mg/dL)	55.1 ± 16.5
LDL cholesterol (mg/dL)	108.9 ± 28.7
Fasting triglycerides (mg/dL)	102 (79–145)*
Serum free T4 (ng/dL)	1.21 ± 0.18

Mean ± s.d. for normal distributed variables.

* Median (interquartile range) for non-normal distributed variables. ^†^Qualitative variables are expressed as frequencies.

### Induction of hyperthyroidism

Hyperthyroidism in rats was induced by chronic subcutaneous (SC) administration of l-thyroxine (T_4_, 100 µg/day, dissolved in 200 µL of saline; Sigma) for a period of three weeks (21 days), as previously described (Lopez *et al*. 2010, Gonzalez *et al*. 2012, Varela *et al*. 2012). Euthyroid (control) rats were treated with vehicle (saline).

### Intracerebroventricular treatments

Intracerebroventricular (ICV) cannulas were stereotaxi­cally implanted under ketamine/xylazine anesthesia, as previously described (Lopez *et al*. 2008,^ 2010^, Whittle *et al*. 2012, Contreras *et al*. 2014, Martinez de Morentin *et al*. 2014, Alvarez-Crespo *et al*. 2016, Martins *et al*. 2016), using the following coordinates 1.6 mm lateral to bregma, 0.6 mm posterior, 4.5 mm deep from the skull. Rats received a single ICV daily administration of T_3_ (4 ng/day, during 5 days) dissolved in 5 µL of saline.

### Stereotaxic microinjection of T_3_ and viral vectors

Rats were placed in a stereotaxic frame (David Kopf Instruments; Tujunga, CA, USA) under ketamine/xylazine anesthesia. Nuclei-specific injections were delivered via a permanent 28-gauge stainless steel cannula (Plastics One, Roanoke, VA, USA) inserted bilaterally either in the VMH or the arcuate nucleus of the hypothalamus (ARC), following stereotaxic coordinates: (a) for the VMH: −2.8 mm posterior to the bregma, ±0.6 mm lateral to bregma and 10.1 mm deep from the skull; (b) for the ARC: −2.8 mm posterior to the bregma, ±0.3 mm lateral to bregma and 10.2 mm deep from the skull. A catheter tube was connected from each infusion cannula to an osmotic minipump flow moderator (Model 1007D; Alzet Osmotic Pumps, Cupertino, CA, USA). These pumps had a flow rate of 0.5 µL/h during 7 days of treatment. The osmotic minipumps were inserted in a subcutaneous pocket on the dorsal surface created using blunt dissection (Imbernon *et al*. 2013, Contreras *et al*. 2014, Martins *et al*. 2016).

Adenoviral GFP or constitutive active AMPKα isoforms (AMPKα-CA; Viraquest; North Liberty, IA, USA) vectors (Woods *et al*. 2000, Minokoshi *et al*. 2004, Lopez *et al*. 2008, 2010) were delivered in the VMH of rats using a 25-gauge needle (Hamilton; Reno, NV, USA) and the stereotaxic coordinates: −2.4 mm and −3.2 mm posterior to the bregma, ±0.6 mm lateral to bregma and 10.1 mm deep at a rate of 200 nL/min for 5 min for rat (1 µL/injection site) as previously reported (Lopez *et al*. 2008, 2010, Martinez de Morentin *et al*. 2012, 2014, Whittle *et al*. 2012, Beiroa *et al*. 2014, Contreras *et al*. 2014, Martins *et al*. 2016). Animals were treated for 6 days.

### Blood biochemistry

For the rat samples, plasma levels of T_3_ and T_4_ were measured using rat ELISA kits (Crystal Chem Inc; Downers Grove, IL, USA) (Lopez *et al*. 2010, Gonzalez *et al*. 2012, Varela *et al*. 2012). For the human samples, serum glucose concentrations were measured in duplicate by the glucose oxidase method using a Beckman Glucose Analyser II (Beckman Instruments; Brea, CA, USA). Roche Hitachi Cobas c711 instrument (Roche) was used to perform HDL cholesterol and total serum triglycerides determinations. HDL cholesterol was quantified by a homogeneous enzymatic colorimetric assay through the cholesterol esterase/cholesterol oxidase/peroxidase reaction (Cobas HDLC3; Roche). Serum fasting triglycerides were measured by an enzymatic, colorimetric method with glycerol phosphate oxidase and peroxidase (Cobas TRIGL; Roche). LDL cholesterol was calculated using the Friedewald formula. Serum free T_4_ was measured by electrochemiluminescence (Roche Diagnostics) with intra- and inter-assay coefficients of variation less than 5%. Methods have been previously reported (Ortega *et al*. 2015, Gavalda-Navarro *et al*. 2016).

### Sample processing

Rats were killed by cervical dislocation. From each animal, gonadal WAT (gWAT), subcutaneous inguinal WAT (sWAT) or both (only for the euthyroid and hyperthyroid animals) were harvested and immediately frozen in dry ice. Samples were stored at −80°C until further processing. Human adipose tissue samples were obtained from sWAT and vWAT depots during elective surgical procedures (cholecystectomy, surgery of abdominal hernia and gastric bypass surgery) (Ortega *et al*. 2015, Gavalda-Navarro *et al*. 2016). Samples of adipose tissue were immediately transported to the laboratory (5–10 min). Tissue handling was carried out under strictly aseptic conditions. Adipose tissue samples were washed in PBS, cut off with forceps and scalpel into small pieces (100 mg), and immediately flash-frozen in liquid nitrogen before storage at −80°C.

### Real-time PCR

We performed real-time PCR (TaqMan; Applied Biosystems) as previously described (Lopez *et al*. 2010, Martinez de Morentin *et al*. 2012, 2014, Whittle *et al*. 2012, Contreras *et al*. 2014, Alvarez-Crespo *et al*. 2016, Martins *et al*. 2016), using specific sets of primers and probes for rat (Supplementary Table 1, see section on supplementary data given at the end of this article). Values were expressed relative to hypoxanthine–guanine phosphoribosyltransferase (HPRT) levels. For the analysis of the human WAT samples, we used commercially available and pre-validated TaqMan primer/probe sets (Applied Biosystems) as follows: endogenous control peptidylprolyl isomerase A (cyclophilin A) (*PPIA*, 4333763), PR domain containing 16 (*PRDM16*, Hs00223161_m1), uncoupling protein 1 (*UCP1*, Hs00222453_m1) and cell death-inducing DFFA-like effector a (CIDEA, Hs00154455_m1). Gene expression values were expressed relative to PPIA levels.

### Histology and immunohistochemistry

Adipose tissue depots were fixed in 10% buffered formaldehyde. For the hematoxylin–eosin processing, the WAT sections were first stained with hematoxylin for 5 min, washed and stained again with eosin for 1 min. The detection of UCP1 in WAT was performed using anti-UCP1 (1:500; ab10983; Abcam) as previously reported (Alvarez-Crespo *et al*. 2016, Martins *et al*. 2016). The specificity of the UCP1 antibody has been previously validated by using WAT samples from UCP1 KO mice (Alvarez-Crespo *et al*. 2016). Images were taken with a digital camera Olympus XC50 (Olympus Corporation) at 20×. Digital images from WAT for immunohistochemistry were quantified with FRIDA image analysis software (FRIDA Software; The Johns Hopkins University; MD, USA); briefly, a color mask (pixel threshold masks) was set to define the UCP1 staining. This color mask was applied to all photographs, and the software obtained a numeric value proportional to the color level in each image. These values are represented with respect to control (100%). For the adipocyte area, images were analyzed with ImageJ Software (National Institutes of Health; MD, USA). Direct detection of GFP fluorescence was performed after perfusion of the animals and detected with a fluorescence microscope Olympus IX51, at 4×.

### Statistical analysis

For the rat experiments, data are expressed as mean ± s.e.m. (error bars represent s.e.m.), mRNA and protein data were expressed in relation (%) to control (euthyroid, vehicle-treated or GFP) rats. Statistical significance was determined by Student’s t-test when two groups were compared or *ANOVA* followed by two-tailed Bonferroni *post hoc* test when more than two groups were compared. *P* < 0.05 was considered statistically significant. For the human studies, statistical analyses were performed using SPSS 12.0 software (IBM). Descriptive results of continuous variables are expressed as mean and s.d. for Gaussian variables or median and interquartile range unless otherwise stated. Parameters that did not fulfill normal distribution were mathematically Log transformed to improve symmetry for subsequent analyses. The relation between variables was analyzed by simple correlation (Pearson’s test and Spearman’s test) and by multivariate regression analysis. Levels of statistical significance were set at *P* < 0.05.

## Results

### Hyperthyroidism induces browning of WAT in rats

**Figure 1:**
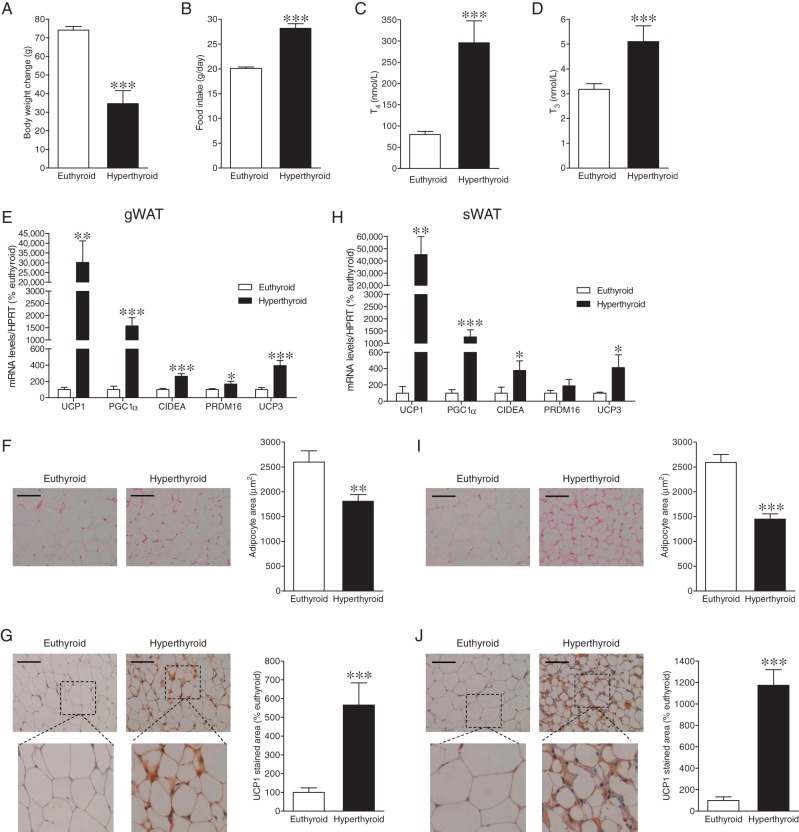
Effect of hyperthyroidism on WAT browning. (A) Body weight change, (B) daily food intake, (C) T_4_ and (D) T_3_ circulating levels of euthyroid and hyperthyroid rats. (E and H) mRNA expression of browning markers, (F and I) representative H&E staining (left panels; 20×, scale bar: 100 μm) and adipocyte area (right panels), and (G and J) representative immunohistochemistry with anti-UCP1 antibody showing UCP1 staining (left panels; 20×, scale bar: 100 μm), UCP1 stained area (right panels) in gWAT and sWAT of euthyroid and hyperthyroid rats. Statistical significance was determined by Student’s *t*-test. *N* = 7 (only for the IHC analyses)-10 animals per group. Error bars represent s.e.m. *, ** and ***P < 0.05, 0.01 and 0.001 vs euthyroid.

T_4_-treated rats exhibited decreased weight gain (Fig. 1A) despite hyperphagia (Fig. 1B). Increased circulating levels of T_4_ (Fig. 1C) and T_3_ (Fig. 1D), confirmed their hyperthyroid status. Next, we analyzed whether hyperthyroidism induced browning of WAT in these animals. Our mRNA data showed that the mRNA expression of browning markers, such as UCP1, peroxisome proliferator-activated receptor gamma coactivator 1-alpha (PGC1α), CIDEA, PRDM16 and also of uncoupling protein 3 (UCP3) was significantly increased in the gWAT (Fig. 1E) and sWAT (Fig. 1H) of hyperthyroid rats. Histological analysis of WAT showed that hyperthyroid rats exhibited a ‘brown-like’ multilocular pattern, associated with decreased adipocyte area (Fig. 1F and I) and increased UCP1 immunostaining (Fig. 1G and J) in both gWAT and sWAT.

### Central T_3_ induces browning of WAT in rats

**Figure 2:**
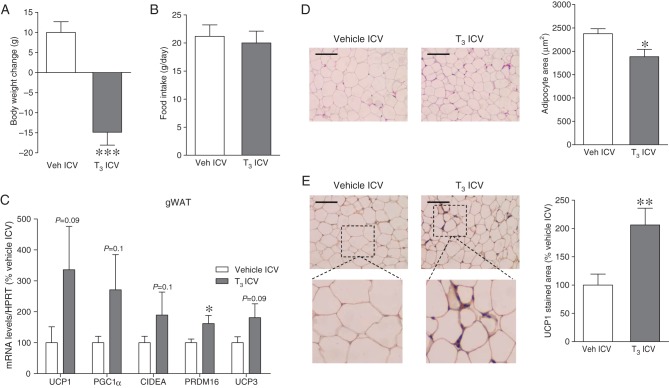
Effect of central T_3_ administration on WAT browning. (A) Body weight change, (B) daily food intake, (C) mRNA expression of browning markers (D) representative H&E staining (left panels; 20×, scale bar: 100 μm) and adipocyte area (right panels), and (E) representative immunohistochemistry with anti-UCP1 antibody showing UCP1 staining (left panels; 20×, scale bar: 100 μm), UCP1 stained area (right panels) in gWAT of vehicle- or T_3_ ICV-treated rats. Statistical significance was determined by Student’s *t*-test. *N* = 7 (only for the IHC analyses)-14 animals per group. Error bars represent s.e.m. *P < 0.05, **P < 0.01, ***P < 0.001 vs vehicle.

Recent data have shown that the effect of THs on thermogenesis is centrally mediated (Lopez *et al*. 2010, Alvarez-Crespo *et al*. 2016). Therefore, we hypothesized that central chronic exposure of T_3_ may stimulate browning of WAT. ICV T_3_ administration induced a feeding-independent decrease in body weight (Fig. 2A and B). mRNA analysis of gWAT showed tendencies even though statistically non-significant for browning markers to be increased (Fig. 2C). Nevertheless, and more relevant, when histological analyses were assessed, our results were much clearer, indicating that ICV T_3_-treated rats displayed a ‘brown-like’ multilocular pattern, associated to decreased adipocyte area (Fig. 2D) and increased UCP1 immunostaining (Fig. 2E).

### T_3_ in the VMH, but not in the ARC, induces browning of WAT in rats

**Figure 3:**
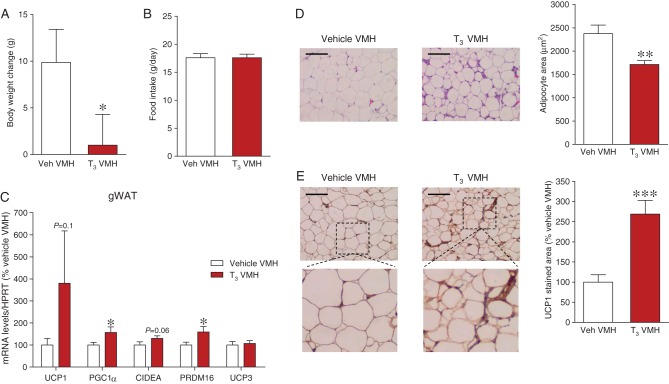
Effect of T_3_ in the VMH on WAT browning. (A) Body weight change, (B) daily food intake, (C) mRNA expression of browning markers, (D) representative H&E staining (left panels; 20×, scale bar: 100 μm) and adipocyte area (right panels), and (E) representative immunohistochemistry with anti-UCP1 antibody showing UCP1 staining (left panels; 20×, scale bar: 100 μm), UCP1 stained area (right panels) in gWAT of vehicle- or T_3_-treated rats in the VMH. Statistical significance was determined by Student’s *t*-test. *N* = 7 (only for the IHC analyses)-18 animals per group. Error bars represent s.e.m. *, ** and ***P < 0.05, 0.01 and 0.001 vs vehicle.

Next, we aimed to identify the hypothalamic nucleus where T_3_ exerted its action on WAT. Therefore, we performed chronic stereotaxic administration of T_3_ into the VMH and the neighboring ARC. The correct position of the cannulae was verified by histological examination of coronal sections of the brains (data not shown). When given into the VMH, T_3_ promoted a feeding-independent weight loss (Fig. 3A and B). On the other hand, when T_3_ was administered into the ARC, there was a tendency to increase body weight at the end of the treatment, which was associated with hyperphagia (Supplementary Fig. 1A and B). mRNA analysis of gWAT showed significantly increased or clear trends toward increased levels of browning markers when T_3_ was delivered within the VMH (Fig. 3C), but not the ARC (Supplementary Fig. 1C). Again, histological analyses confirmed that VMH T_3_-treated rats displayed decreased adipocyte area (Fig. 3D) and increased UCP1 immunostaining (Fig. 3E) in gWAT, confirming browning.

### Central effects of T_3_ on browning of WAT depend on AMPK in the VMH

**Figure 4:**
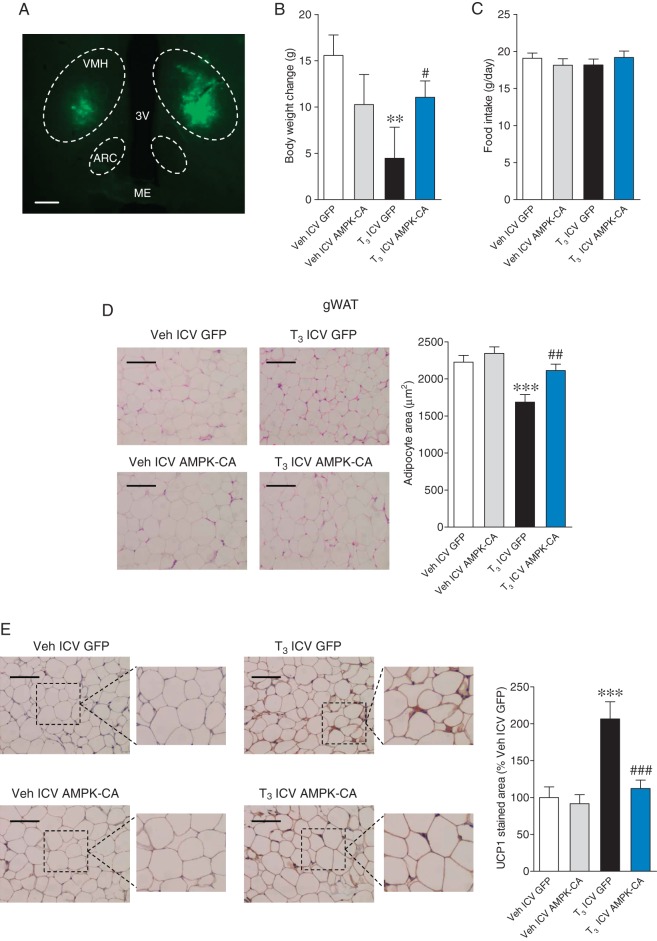
Effect of AMPK overexpression in the VMH and central T_3_ administration on WAT browning. (A) Bilateral GFP fluorescence in VMH (4×, scale bar 100 μM), (B) body weight change, (C) daily food intake, (D) representative H&E staining (left panels; 20×, scale bar: 100 μm) and adipocyte area (right panels), and (E) representative immunohistochemistry with anti-UCP1 antibody showing UCP1 staining (left panels; 20×, scale bar: 100 μm), UCP1-stained area (right panels) in gWAT of rats stereotaxically treated with GFP or AMPKα-CA adenovirus and ICV treated with vehicle or T_3_. Statistical significance was determined by ANOVA. *N* = 8 (only for the IHC analyses)-10 animals per group. Error bars represent s.e.m. ** and ***P < 0.01 and 0.001 vs vehicle ICV GFP; ^#^, ^##^ and ^###^*P* < 0.05, 0.01 and 0.001 vs T_3_ ICV GFP.

Next, we investigated the molecular mechanisms within the VMH leading to modulation of browning after central T_3_ administration. Recent evidence has linked the inhibition of hypothalamic AMPK, and more specifically within the VMH, as a mechanism for the central regulation of BAT thermogenesis by THs (Lopez *et al*. 2010,^ 2016^, Alvarez-Crespo *et al*. 2016). Based on this evidence, we hypothesized that the central effect of T_3_ on browning might be mediated by specific inhibition of AMPK in the VMH. To test this, adenoviruses encoding either a constitutively active isoform of AMPKα (AMPKα-CA) or a GFP control vector were injected stereotaxically into the VMH of ICV T_3_-treated rats. The AMPKα-CA adenovirus was previously validated (Lopez *et al*. 2010, Martinez de Morentin *et al*. 2012, 2014, Whittle *et al*. 2012, Beiroa *et al*. 2014, Martins *et al*. 2016). Overexpression of AMPKα-CA in the VMH, confirmed by GFP immunofluorescence (Fig. 4A), blunted the weight loss caused by central T_3_ injection, without alteration in feeding (Fig. 4B and C). Of note, this effect was associated with the reversal of the T_3_-induced browning of gWAT, as demonstrated by increased adipocyte area (Fig. 4D) and decreased UCP1 staining (Fig. 4E) in T_3_-treated rats receiving AMPKα-CA adenoviruses in the VMH compared with those treated with control GFP adenoviruses. Together, these results are consistent with the observation that AMPK activity in the VMH mediates the central effects of T_3_ on browning of WAT.

### Browning markers in WAT are positively correlated with circulating T_4_ in humans

**Figure 5:**
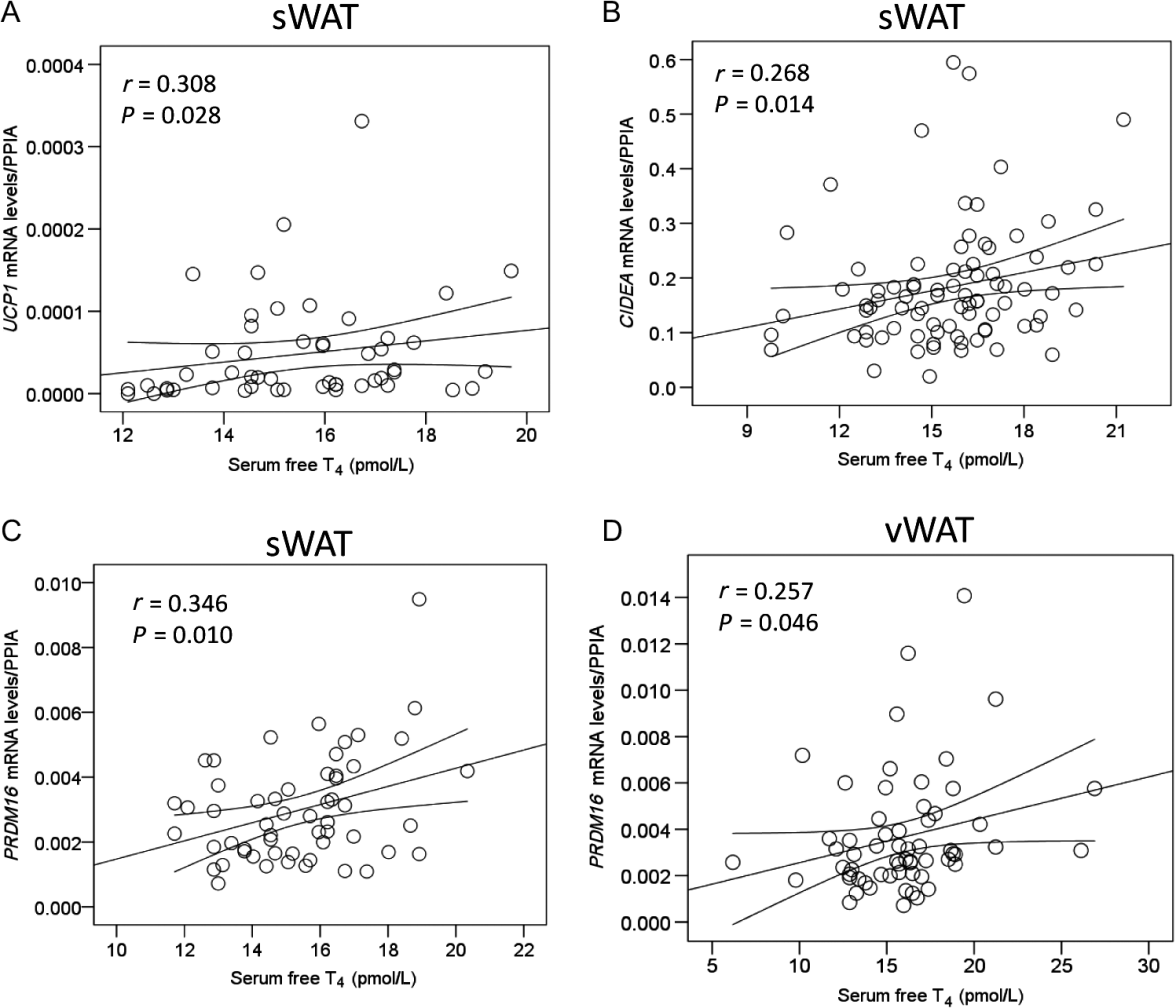
Correlation between T_4_ circulating levels and browning markers in human WAT. Correlation between *UCP1* (A), *CIDEA* (B) and *PRDM16* (C) in sWAT and *PRDM16* in vWAT (D) in human subjects.

Finally, we analyzed the relationship between T_4_ serum concentrations and the mRNA expression levels of browning marker in sWAT and vWAT, in samples derived from a large cohort of patients. Our data showed that the mRNA levels of UCP1 and CIDEA in sWAT (Fig. 5A and B, Supplementary Fig. 2) and PRDM16 in sWAT and vWAT (Fig. 5C and D), correlated with circulating free T_4_, showing a positive association between THs and browning in humans. Multivariate regression analysis indicated that serum free T_4_ levels contributed significantly to browning-related (PRDM16, CIDEA and UCP1) mRNA levels variation after controlling for age, gender and BMI (Table 2).

**Table 2:** Multivariate regression analyses to predict sWAT *CIDEA*, *PRDM16* and *UCP1* and vWAT *PRDM16* gene expression in the human cohort.

	sWAT CIDEA		sWAT PRDM16		sWAT UCP1		vWAT PRDM16	
	β	P	β	P	β	P	β	P
Age (years)	−0.16	0.15	−0.14	0.28	0.08	0.6	0.15	0.25
Sex	−0.05	0.63	−0.01	0.92	0.14	0.38	−0.12	0.34
BMI (kg/m2)	−0.21	**0.04**	−0.17	0.19	0.05	0.76	−0.27	**0.03**
Serum free T4 (pmol/L)	0.24	**0.03**	0.35	**0.01**	0.4	**0.02**	0.24	0.05
Adjusted R2	0.096 (9.6%)		0.105 (10.5%)		0.087 (8.7%)		0.101 (10.1%)	
P (model)	**0.018**		**0.04**		0.1		**0.4**	

*β* corresponds to the standardized beta coefficient of the multiple regression analyses. Bold indicates statistical significance.

## Discussion

In this study, we show that THs induce browning of WAT in rodents and circulating T_4_ levels correlate with the expression of browning markers in the WAT of humans. The effect of THs is centrally mediated involving specifically the VMH, a key nucleus modulating energy balance (Morrison *et al*. 2014, Contreras *et al*. 2015, Lopez *et al*. 2016). Notably, this hypothalamic mechanism is mediated through AMPK, which has been described as a key factor regulating the actions of THs at the central level (Lopez *et al*. 2010, 2016, Alvarez-Crespo *et al*. 2016).

It has been known for more than a century that THs increase the basal metabolic rate (Magnus-Levy 1895). Typically, most of these effects have been related to the direct actions of THs on metabolically active tissues, such as the liver (Yen 2001), BAT (Bianco *et al*. 2005, Lopez *et al*. 2010, Ribeiro *et al*. 2010, Alvarez-Crespo *et al*. 2016), heart (Klein & Ojamaa 2001, Kahaly & Dillmann 2005) and skeletal muscle (Short *et al*. 2001). In those tissues, THs increase metabolic rate and thermogenesis by promoting the generation of energy and also by reducing the thermodynamic efficiency, which lead to heat production and increased temperature (Hulbert & Else 1981, Silva 2006, Lopez *et al*. 2013).

The process in which precursor cells placed in WAT become beige/brite cells, instead of white adipocytes, is called browning (Fisher *et al*. 2012, Shabalina *et al*. 2013). Consequently, certain WAT depots significantly increase gene expression for UCP1 and their thermogenic capacity (Shabalina *et al*. 2013). Although the sWAT from the inguinal area is the most classical fat pad where browning studies have been performed, it has also been described in other depots, such as gonadal (Plum *et al*. 2007, Tews *et al*. 2013, Neinast *et al*. 2015, Contreras *et al*. 2016*a*, Fulzele *et al*. 2016, Jia *et al*. 2016, Lee *et al*. 2016, Martins *et al*. 2016, Shao *et al*. 2016). In this sense, it has been recently demonstrated that when centrally induced, browning affects gWAT in a similar extent to inguinal sWAT (Contreras *et al*. 2016*a*, Martins *et al*. 2016). However, despite the main thermogenic role of central THs, whether they are able to modulate the browning of WAT remains unclear. Here, we show that hyperthyroidism induces browning of WAT (sWAT from the inguinal area and gWAT) in rats. In our hyperthyroid model, T_4_ was administered peripherally, which might imply the existence of direct effects of THs on white adipocytes, known to express TRs (Brent 2012, Lopez *et al*. 2013). Alternatively, THs may exert a central action after crossing the blood–brain barrier (BBB), which would be in agreement with recent evidence from our group, demonstrating that the metabolic effects of THs on brown fat are centrally mediated (Lopez *et al*. 2010, Alvarez-Crespo *et al*. 2016). Therefore, we investigated the contribution of the central effects of THs on browning of WAT. Our data show that, when administered centrally, T_3_ promotes a similar pattern of browning of WAT as observed in the hyperthyroid model. Remarkably, the central action of T_3_ targets one particular hypothalamic nucleus, the VMH. Indeed, stereotaxic administration of the hormone into the ARC (a neighboring nucleus) did not recapitulate the effects on the browning program induced by T_3_ within the VMH. Considering that the VMH also plays a major role in the modulation of BAT function via THs (Lopez *et al*. 2010,^ 2013^, ^2016^, Alvarez-Crespo *et al*. 2016), our data suggest that this hypothalamic site is a key modulator of both white and brown fat activity.

Current evidence has demonstrated that inhibition of AMPK in the VMH plays a major role in mediating either the actions of THs on BAT (Lopez *et al*. 2010, 2013, 2016, Alvarez-Crespo *et al*. 2016) or the browning of WAT, for example by glucagon-like peptide 1 (GLP-1) agonism (Beiroa *et al*. 2014). To elucidate the contribution of hypothalamic AMPK activity on the browning of WAT by THs, we genetically activated AMPK in the VMH of rats centrally treated with T_3_. Our data showed that activation of AMPK totally blunted the effects of T_3_ on WAT browning. Interestingly, this action was associated with a feeding-independent recovery of body mass, which was reduced by central T_3_. This evidence suggests that the augmented thermogenic capacity of brite adipocytes (Shabalina *et al*. 2013) participates together with the BAT-mediated action in the weight-reducing effects of central T_3_.

Finally, we aimed to investigate whether browning markers correlate with circulating THs levels in humans. Remarkably, our results indicate that serum levels of T_4_ are positively associated with *UCP1*, *CIDEA* and *PRDM16* in WAT. To our knowledge, this is the first demonstration that THs modulate WAT browning in humans. Whether increased WAT browning is observed in hyperthyroid patients is not reported, but considering that THs also stimulate BAT in humans (Lahesmaa *et al*. 2014) and that most of the human BAT is actually beige fat (Jespersen *et al*. 2013, Shinoda *et al*. 2015), it is tempting to speculate that browning of WAT may account for the increased energy expenditure that characterizes hyperthyroidism (Warner & Mittag 2012, Lopez *et al*. 2013). In this sense, we have performed some preliminary studies in hyperthyroid patients and detected a trend in the correlation between UCP1 mRNA expression and T_4_; however, further work will be necessary to properly investigate this association. If that is the case, strategies to modulate browning might be of therapeutic benefit in controlling the effects of thyrotoxicosis. This latter possibility is particularly relevant in the context of life-threatening conditions, such as thyroid storm, for which current treatments are not satisfactory. In addition, the induction of browning by TR agonism might be a suitable strategy for the treatment of obesity. In this regard, recent data have shown that treatment with the TR agonist GC-1 promotes browning of WAT and ameliorates obesity and diabetes in mice (Lin *et al*. 2015).

In summary, our results make evident the importance of THs in the browning of WAT in rodent and humans. This observation provides new insights into the physiological effects of THs and also in the pathogenesis of hyperthyroidism-induced effects on energy balance; it also suggests potential therapeutic strategies to counteract this disorder or other catabolic states.

## Supplementary Material

Supporting Figure 1

Supporting Figure 2

Table S1 Primers *and probes for real-time PCR (TaqMan®) analysis*.

## Declaration of interest

The authors declare that there is no conflict of interest that could be perceived as prejudicing the impartiality of the research reported.

## Funding

The research leading to these results has received funding from the European Community’s Seventh Framework Programme (FP7/2007–2013) under grant agreement n° 281854 – the *ObERStress* project (M L), Xunta de Galicia (R N: 2015-CP080 and PIE13/00024; M L: 2015-CP079), MINECO co-funded by FEDER (R N: BFU2015-70664-R; C D: BFU2014-55871-P; M L: SAF2015-71026-R and BFU2015-70454-REDT/*Adipoplast*). CIBER de Fisiopatología de la Obesidad y Nutrición is an initiative of ISCIII. The funders had no role in study design, data collection and analysis, decision to publish or in the preparation of the manuscript.

## Author contribution statement

N M-S, J M M-N, C C and E R-P performed the *in vivo* experiments (hormonal, drug and viral treatments) and the analytical methods (real-time quantitative PCR and immunohistochemistry). N M-S, J M M-N, J F, R N, C D, J M F-R and M L designed the experiments, analyzed, discussed and interpreted the data. N M-S, J M M-N and M L made the figures. M L developed the hypothesis, coordinated and directed the project and wrote the manuscript. All authors reviewed and edited the manuscript and had final approval of the submitted manuscript.
