# Supplementary figures and images for: Thyroid hormones induce browning of white fat
Source: J Endocrinol. 2016 Dec 2;232(2):351–62. doi: 10.1530/JOE-16-0425 (PMC5292977; doi:10.1530/JOE-16-0425)

**Suppl. Figure 1**  
(Martínez-Sánchez et al

**A**

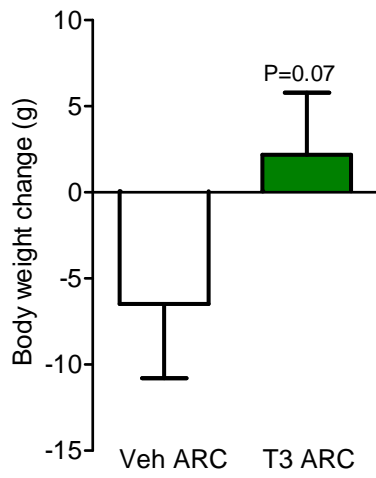

**B**

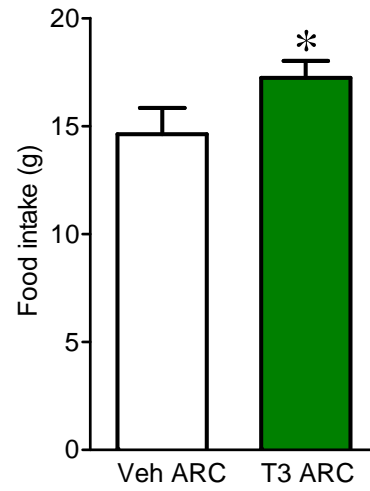

**C**

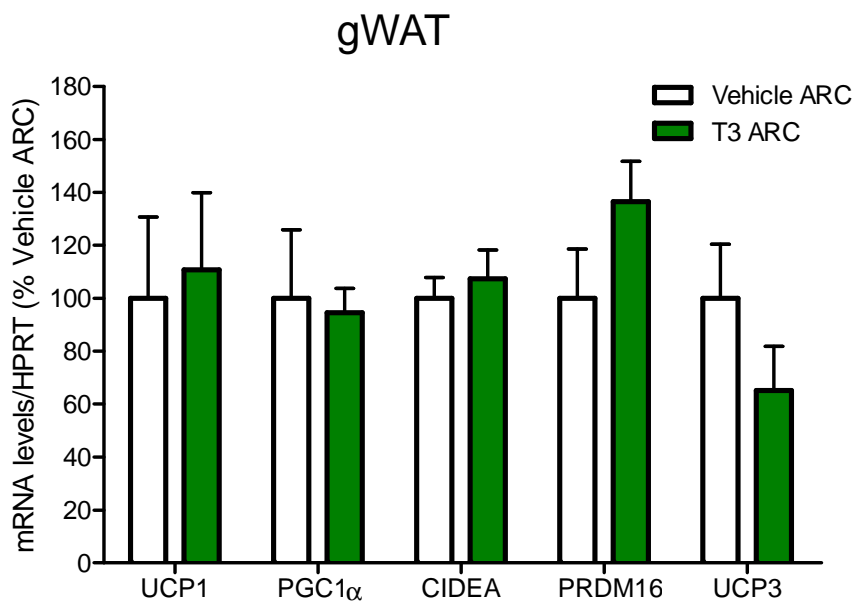

Supplement: Supporting Figure 1 [file joe-232-351-s001.pdf]

**Suppl. Figure 2**  
(Martínez-Sánchez et al

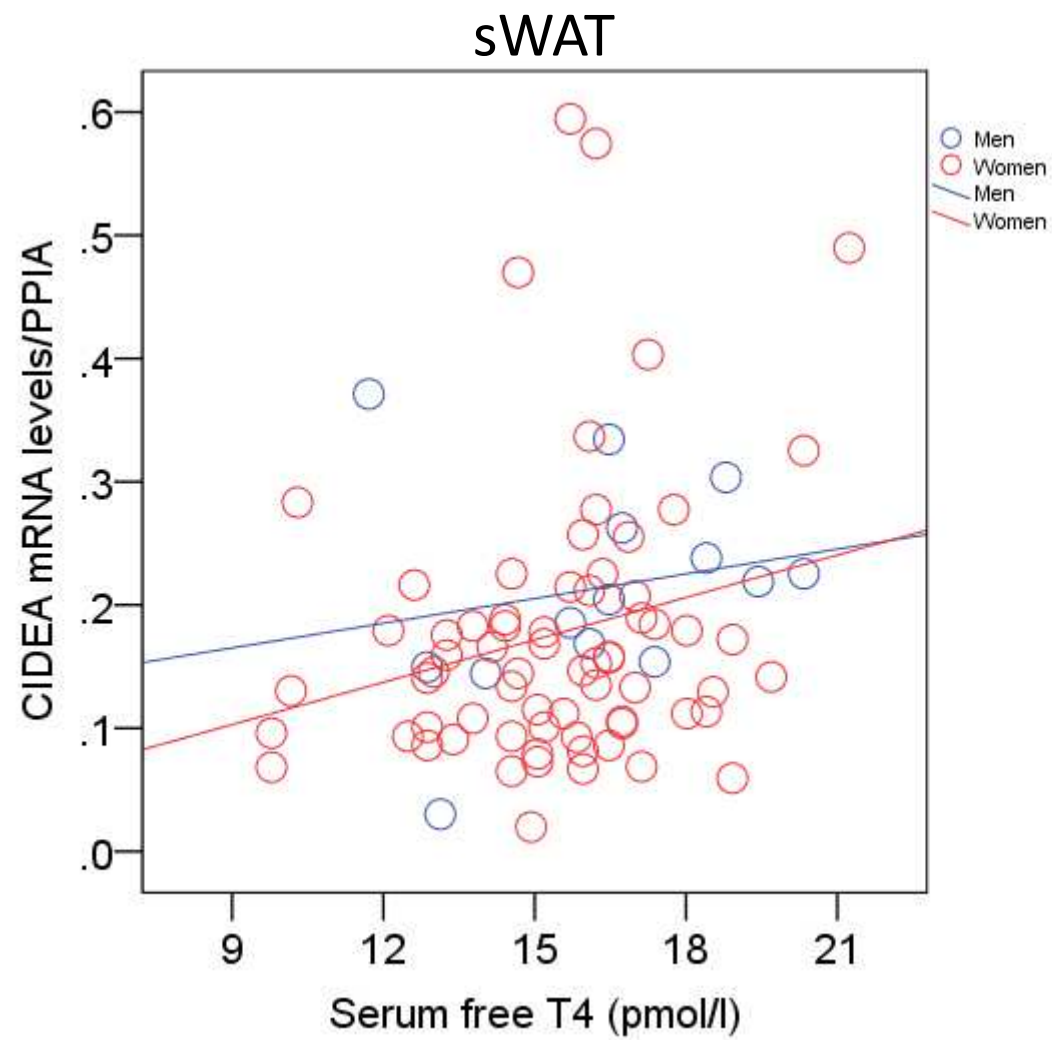

Supplement: Supporting Figure 2 [file joe-232-351-s002.pdf]
