# Supplementary material for: Thyroid hormones induce browning of white fat
Source: J Endocrinol. 2016 Dec 2;232(2):351–62. doi: 10.1530/JOE-16-0425 (PMC5292977; doi:10.1530/JOE-16-0425)
Supplement: Table S1 [file joe-232-351-t001.pdf]

Supplemental Table 1. Primers *and probes for real-time PCR (TaqMan®) analysis.*

mRNA	GenBank Accession Number		Sequence
**CIDEA**	NM_001170467.1	Assay ID	ThermoFisher TaqMan® Gene Expression Assays<br>Assay ID Rn04181355_m1
**HPRT**	NM_012583	Fw Primer	5’-AGCCGACCGGTTCTGTCAT-3'
		Rv Primer	5’-GGTCATAACCTGGTTCATCATCAC -3'
		Probe	FAM-5’- CGACCCTCAGTCCCAGCGTCGTGAT 3’-TAMRA
**PGC1α**	NM_031347	Fw Primer	5’-CGATCACCATATTCCAGGTCAAG-3’
		Rv Primer	5’-CGATGTGTGCGGTGTCTGTAGT -3’
		Probe	FAM-5’-AGGTCCCCAGGCAGTAGATCCTCTTCAAGA -3’-TAMRA
**PRDM16**	XM_008764418.1	Assay ID	ThermoFisher TaqMan® Gene Expression Assays<br>Assay ID Mm01266512_m1
**UCP1**	NM_012682	Fw Primer	5’-CAA TGA CCA TGT ACA CCA AGG AA-3’
		Rv Primer	5’-GAT CCG AGT CGC AGA AAA GAA-3’
		Probe	FAM-5’-ACC GGC AGC CTT TTT CAA AGG GTT TG-3’-TAMRA
**UCP3**	NM_003356.3	Assay ID	ThermoFisher TaqMan® Gene Expression Assays<br>Assay ID Rn00565874_m1
